# LncRNA Profiling and ceRNA Network Construction of Intrauterine Exosomes in Goats During Embryo Implantation

**DOI:** 10.3390/ani15172471

**Published:** 2025-08-22

**Authors:** Yanni Jia, Huixin Zhang, Wei Wang, Zuhui Li, Chunmei Shang, Haokun Liu, Hongyu Niu, Dong Zhou, Yaping Jin, Pengfei Lin

**Affiliations:** 1 College of Veterinary Medicine, Northwest A&F University, Yangling, Xianyang 712100, China; ynj@nwafu.edu.cn (Y.J.);; 2 Key Laboratory of Animal Biotechnology, Ministry of Agriculture and Rural Affairs, Northwest A&F University, Yangling, Xianyang 712100, China

**Keywords:** goat, embryo implantation, lncRNA, exosome, uterine flushing fluid

## Abstract

Exosomes have been shown to play an important role in embryo implantation, but the mechanism is still unclear. This study aimed to investigate the functional roles of lncRNAs in intrauterine exosomes in goat pregnancy. We used RNA-seq to identify the lncRNA profiles of exosomes obtained from goat uterine rinsing fluid at 5, 15, and 18 days of gestation. In addition, we performed weighted gene co-expression network analysis based on differentially expressed mRNAs (DEMs) and lncRNAs (DELs). Functional enrichment analyses of gene modules were conducted using Gene Ontology classification (GO) and Kyoto Encyclopedia of Genes and Genomes (KEGG) pathway. A lncRNA-miRNA-mRNA competing endogenous RNA (ceRNA) regulatory network was constructed based on predictive interaction derived from miRTarBase, miRDB and RNAhybrid databases. Altogether, 831 DELs were identified. GO and KEGG analysis showed that the target genes were enriched in processes associated with embryo implantation, such as signaling receptor activity, binding and immune response. Nine functional co-expression modules were enriched in various biological processes, such as metabolic pathways, protein transport, cell cycle and VEGF signaling pathway. Additionally, 12 lncRNA-mediated ceRNA networks were constructed. Our results demonstrate that exosomal lncRNAs in uterine flushing fluid exhibit dynamic changes across gestational stages and play an important role in regulating the uterine microenvironment during embryo implantation. These findings provide a foundational basis for screening exosome-derived lncRNAs that influence embryo implantation and contribute to elucidating the mechanistic roles of lncRNAs in exosome-mediated processes during early pregnancy.

## Simple Summary

Exosomes are critical for embryo implantation, but their mechanisms remain unclear. This study characterized lncRNAs in uterine flushing fluid-derived exosomes during goat pregnancy (days 5, 15, 18 of gestation) via RNA-seq. We identified 831 differentially expressed lncRNAs across stages, enriched in early pregnancy-related processes (e.g., cell communication, immune modulation). Further analysis revealed dynamic temporal changes in lncRNA and mRNA expression patterns in uterine exosomes across gestational stages. Additionally, we constructed competing endogenous RNA regulatory networks centered on key differentially expressed lncRNAs. These findings provide a solid basis for screening and studying implantation-regulating exosomal lncRNAs in goats.

## 1. Introduction

Embryo implantation is a critical step in establishing pregnancy in ruminants. During this process, the embryo undergoes proliferation, migration, positioning, adhesion, and invasion to form a tight junction with the maternal endometrium. The success of embryo implantation determines the outcome of pregnancy, and it primarily depends on two factors: the embryo and the establishment of uterine receptivity [1]. Studies have shown that 75% of embryo loss occurs during the pre- and post-implantation periods. [2]. Therefore, elucidating the regulatory mechanisms of embryo implantation has great significance for improving mammalian reproductive rates.

Uterine flushing fluids (UFs) support embryo development and survival during the embryo implantation stage [3]. UFs contain a complex milieu of nutrients, growth factors, and extracellular vesicles (EVs), which facilitate intercellular signaling and nutrient transport [4]. EVs have gained significant attention due to their role as mediators of cell-to-cell communication: they carry bioactive molecules (e.g., proteins, lipids, mRNAs, non-coding RNAs [ncRNAs]) and can transfer functional cargo to target cells, modulating processes such as proliferation, apoptosis, and immune regulation [5,6]. EVs in UFs have been identified and found to be involved in embryo development and implantation. For instance, intrauterine EVs containing miR-98 regulate maternal immune responses during the peri-implantation period in cattle [7], while bta-miR-26b-enriched EVs in uterine fluids suppress maternal immunity to facilitate conceptus implantation into the endometrium [8]. Multi-omics sequencing analyses further reveal that uterine fluid-derived EVs undergo dynamic transcriptomic changes throughout the uterine implantation window, underscoring their adaptive role in supporting embryo development [9,10].

With advancements in high-throughput sequencing technology, it has been determined that the regulation of biological processes relies not only on mRNA but also on non-coding RNAs, including miRNA, circular RNA, and lncRNA. LncRNAs, defined as transcripts exceeding 200 nucleotides in length, are characterized by low evolutionary conservation, tissue-specific expression patterns, and generally low abundance. Substantial research has established lncRNAs as key regulators of cellular processes, including protein-coding gene translation, signaling pathway modulation, and epigenetic regulation, with diverse biological functions across physiological and pathological contexts [11,12]. Among these, lncRNAs and mRNAs regulate downstream target gene expression by competing for shared sequences in miRNAs, known as the competing endogenous RNA (ceRNA) network. Currently, ceRNA networks have been confirmed to be involved in various biological processes, including different cancers and reproductive diseases [13,14,15,16]. In ruminants, ceRNA networks have also been implicated in early pregnancy regulation. For instance, lncRNA882 in goat endometrial epithelial cells modulates leukemia inhibitory factor (LIF) expression via miR-15b, thereby influencing endometrial receptivity establishment [17]. Additionally, ceRNA networks associated with reproduction in sheep have been characterized, underscoring their involvement in pregnancy regulation [18]. Nevertheless, current research remains limited, and the regulatory networks and mechanistic roles of exosome-derived lncRNAs in early pregnancy of ruminants warrant further exploration.

In this study, we isolated exosomes from goat UFs at 5, 15, and 18 days of gestation and used RNA-seq to identify the lncRNAs profiles of exosomes obtained from UFs during embryo implantation. Following the identification of differentially expressed lncRNAs, we integrated these with previously identified differentially expressed miRNAs and mRNAs from the corresponding gestational stages (generated by our laboratory) and performed comprehensive bioinformatics analyses to construct co-expression modules and ceRNA regulatory networks [19]. These results will help to further elucidate the role of exosomes in early pregnancy and provide new insights into the interaction mechanisms between lncRNAs and mRNAs during embryo implantation.

## 2. Materials and Methods

### 2.1. Sample Collection and Exosome Isolation

Sample collection, exosome isolation, and identification have been reported in previous publications [19]. In brief, Guanzhong dairy goats kept in the Yangling Northwest Agriculture and Forestry University experimental animal center were split into three groups at random, and collecting UFs on day 5 (D5P, in the pre-implantation period), day 15 (D15P, in the pregnancy recognition period) and day 18 (D18P, in the embryo adhesion period) of pregnancy that was determined by looking at the tubular or linear pregnancy in the uterus. UFs were centrifuged at 12,000× *g* for 45 min at 4 °C to remove debris; supernatants were collected. The supernatants were filtered through 0.22 μm membranes, and filtrates ultracentrifuged at 110,000× *g* for 70 min at 4 °C. After discarding supernatants, exosomes were resuspended. Exosome identification employed three methods: (1) Transmission Electron Microscopy (TEM) (Tecnai G2 Spirit BioTWIN, FEI, Hillsboro, OR, USA): Exosomes (5 μL) from ultracentrifugation were diluted to 10 μL, applied to a copper grid, settled for 1 min, blotted, stained with 10 μL phosphotungstic acid for 1 min, dried, and observed under an electron microscope (HITACHI, Tokyo, Japan) (80 kV) to visualize morphology. (2) Nanoparticle Tracking Analysis (NTA): Diluted exosomes (30 μL) were calibrated and analyzed via NanoFCM (NanoFCM, Xiamen, China) to determine particle size distribution and concentration. (3) Western Blot: Total exosomal proteins were extracted using the Exosome Protein Extraction Kit (KeyGEN BioTECH, Nanjing, China), quantified with the BCA Protein Assay Kit (KeyGEN BioTECH, Nanjing, China), separated by SDS-PAGE, transferred to PVDF membranes, blocked with 5% skim milk for 1 h, incubated overnight at 4 °C with primary antibodies (TSG101/CD63, Abcam, 1:1000, Cambridge, UK), followed by 1 h secondary antibody (ZHHC, 1:5000, Eagan, MN, USA) at room temperature, visualized via ECL, and imaged (Bio-Rad, Hercules, CA, USA) to confirm marker expression.

### 2.2. RNA-Seq Analysis

Total RNA was extracted from exosomes using miRNeasy-Serum/Plasma Kit (Qiagen, Düsseldorf, Germany) following the manufacturer’s protocol. In brief, exosomes were thawed at 37 °C, lysed with 500 µL QIAGEN Lysis Buffer (5 min), mixed with 100 µL chloroform (15 s vortex, 3 min), and centrifuged (12,000× *g*, 15 min). The aqueous phase was combined with 1.5× volumes anhydrous ethanol, loaded onto an RNeasy MinElute column (8000× *g*, 15 s). The column was washed with 700 µL RWT (8000× *g*, 15 s) and centrifuged (12,000× *g*, 5 min). RNA was eluted with 14 µL RNase-free water (12,000× *g*, 1 min). RNA quality assessment and sequencing were performed by BGI Genomics (Shenzhen, China). Raw RNA-seq reads generated in this study have been deposited in the NCBI Sequence Read Archive (SRA) under the BioProject accession number PRJNA1286071.

### 2.3. Identification of Differentially Expressed lncRNAs (DELs)

The cleaned data was compared to the reference gene set using Bowtie2 (v2.3.4.3) [20]. The reference gene set was provided by the Dr. Tom multi-omics data mining system. RSEM (v1.3.1) software was used for gene or transcript expression quantification, and pheatmap (v1.0.8) was used to plot the clustering heatmap of gene expression in different samples [21]. DELs were identified via DESeq2 (v1.20.0), *p*-values were then converted to *q*-values via the two methods proposed by Hochberg, Benjamin, Storey, and Tibshirani, with statistical significance defined as a *q*-value ≤ 0.05 and |log_2_ fold change (log_2_FC)| > 1 [22].

### 2.4. Reverse Transcription Quantitative Polymerase Chain Reaction (RT-qPCR)

Total RNA was extracted following the aforementioned protocol. Reverse transcription was performed using the “Evo M-MLV RT Kit with gDNA Clean for qPCR” (Catalog No. AG11705, Accurate Biotechnology (Hunan) Co., Ltd., Changsha, China) by preparing the reaction system according to the manufacturer’s instructions. Subsequently, RT-qPCR was conducted using the resulting cDNA as the template with the SYBR Green Premix Pro Taq HS qPCR Kit (AG11701, Accurate Biotechnology (Hunan) Co., Ltd., Changsha, China) on a CFX96™ RealTime PCR Detection System (Bio-Rad, Hercules, CA, USA). Target gene expression was quantified using the 2^−ΔΔCt^ method. *GAPDH* was used as a reference gene for normalization. The primers used for RT-qPCR are listed in Table 1.

### 2.5. Construction of Co-Expression Network

A total of 2974 differentially expressed mRNAs (DEMs) were downloaded from the previous sequencing data [19]. And gene co-expression networks were constructed based on DEMs and DELs using the weighted gene co-expression network analysis (WGCNA) package in R software (v1.70) [23].

### 2.6. GO and KEGG Enrichment Analysis

We used RNAplex to predict the target genes of the DELs [24]. GO and KEGG pathway enrichment analyses were conducted using clusterProfiler (v3.8.1). Heatmap was plotted by (https://www.bioinformatics.com.cn) (accessed on 12 May 2025), an online platform for data analysis and visualization.

### 2.7. Construction of ceRNA Network

To construct the ceRNA network, we obtained the predictive interaction of DELs in the WGCNA modules with miRNAs by RNAhybrid (http://bibiserv.techfak.uni-bielefeld.de/rnahybrid) (accessed on 2 January 2025) [25]. Meanwhile, the interaction between DEMs and miRNAs were predicted by miRTarBase (http://mirtarbase.cuhk.edu.cn/) (accessed on 2 January 2025) [26], miRanda (http://www.microrna.org/) (accessed on 2 January 2025) [27]. We screened the differentially expressed miRNAs with the opposite expression pattern of lncRNA and mRNA in the previous sequencing [19]. Then we constructed and visualized the lncRNA-miRNA-mRNA ceRNA network based on lncRNA-miRNA and miRNA-mRNA pairs using the Cytoscape software (version 3.7.0; Cytoscape Consortium, La Jolla, CA, USA).

### 2.8. Statistical Analysis

Data are expressed as mean ± standard error of the man (SEM). One-way analysis of variance (ANOVA) was used to analyze statistical differences. Analysis was performed using GraphPad Prism version 6.01 (GraphPad Software, San Diego, CA, USA). Groups labeled with different letters (e.g., a, b, c) indicate significant differences (*p* < 0.05).

## 3. Results

### 3.1. Identification of DELs

**Figure 1:**
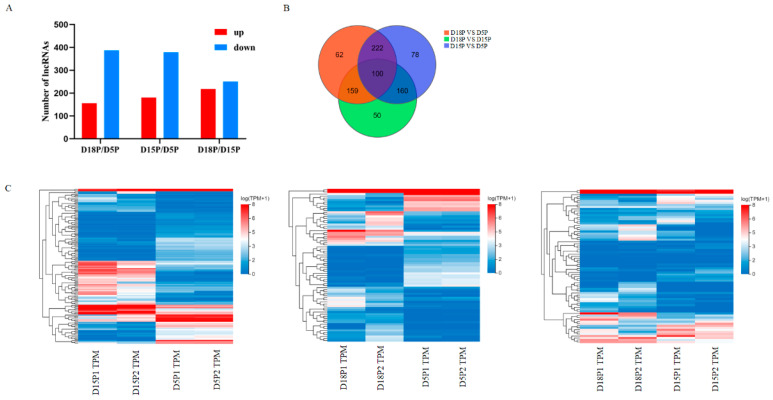
Differential lncRNAs in exosomes isolated from the 5th day, 15th day, and 18th day of pregnancy. (**A**) Differential abundance of lncRNA in groups. (**B**) The Venn diagram shows the DELs. (**C**) Hierarchical clustering heatmaps of DELs.

Using high-throughput sequencing techniques, we evaluated the variations in exosome lncRNAs in uterine flushing fluid at 5, 15, and 18 days of gestation. In this study, 831 DELs were found. Within the D18P vs. D5P comparison group, 156 DELs were significantly up-regulated, and 387 were significantly down-regulated (Figure 1A). Among these DELs, 181 were up-regulated and 379 were down-regulated in the D15P vs. D5P comparison (Figure 1A). Additionally, 218 DELs showed up-regulation, and 251 exhibited down-regulation in the D18P vs. D15P comparison group (Figure 1A). There were 100 DELs across the three groups (Figure 1B). Cluster analysis showed the dynamic changes in the isolated exosomes in goat uterine flushing fluid during different gestation periods (Figure 1C).

### 3.2. GO Enrichment and KEGG Pathway Analysis of Predicted Target Genes of DELs

**Figure 2:**
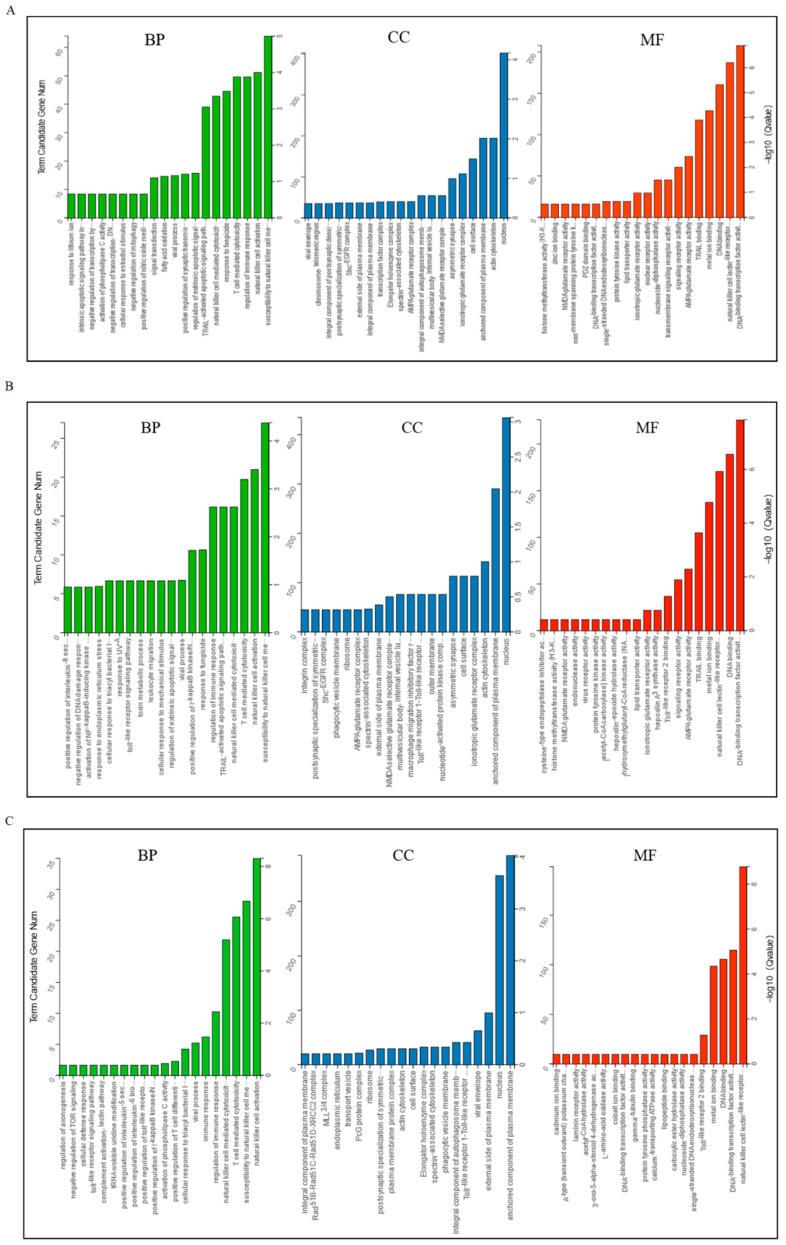
Gene ontology classifications of target genes of DELs in exosomes. (**A**) D5P vs. D15P; (**B**) D5P vs. D18P; (**C**) D15P vs. D18P. The *X*-axis represents enriched terms, the left *Y*-axis represents the number of enriched genes, and the right *Y*-axis represents the *p*-value. BP: biological process; CC: cellular component; MF: molecular function.

Based on the top 60 GO terms, the GO analysis results were assigned to biological processes (BP), cellular component (CC), and molecular function (MF). The GO analysis showed that the target genes were mainly enriched in signaling receptor activity, binding, and immune response (Figure 2).

**Figure 3:**
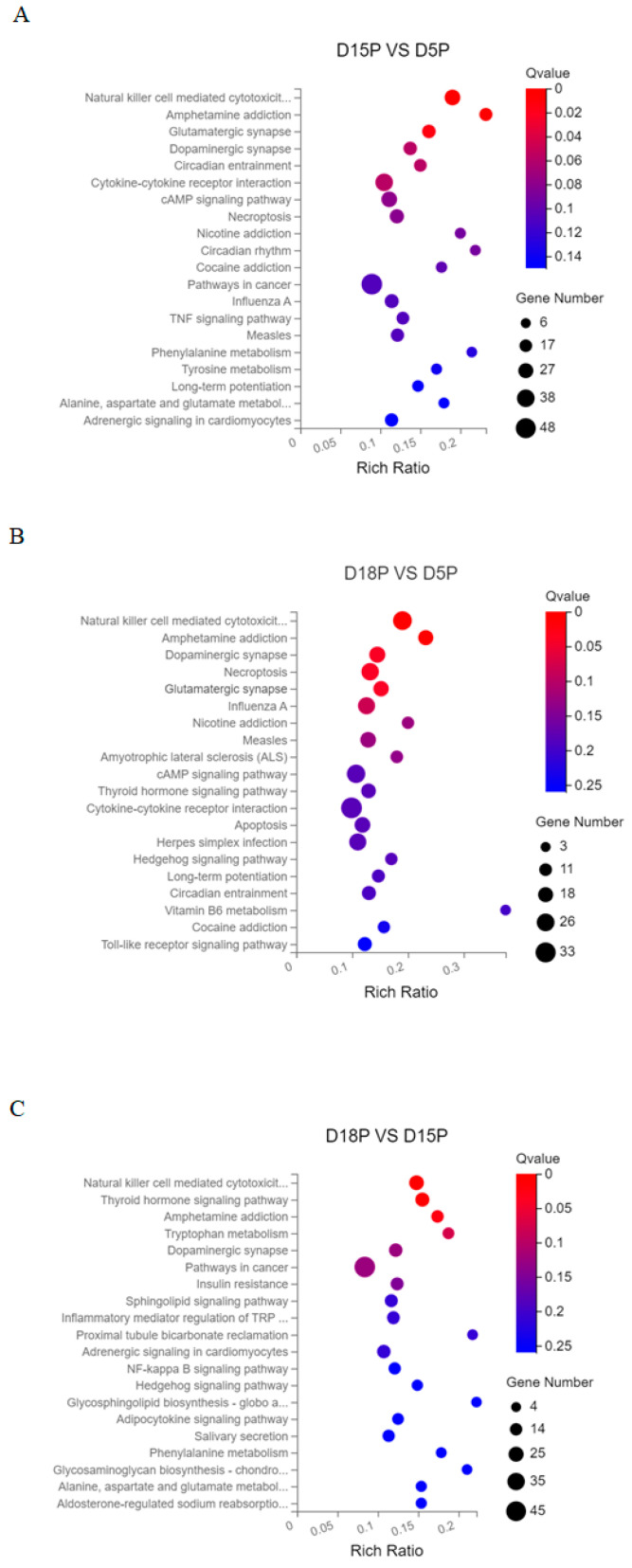
Top 20 KEGG pathways of DEL target genes. (**A**) D5P vs. D15P; (**B**) D5P vs. D18P; (**C**) D15P vs. D18P. The *x*-axis indicates the number of unique sequences assigned to a specific pathway and the *y*-axis indicates the KEGG pathway. The size of the circle represents the number of genes enriched in the pathway.

Using the KEGG database, signal pathway annotation of target genes of DELs was carried out in each group (only the top 20 are shown). “Natural killer cell mediated cytotoxicity”, “Amphetamine addiction”, and “Glutamatergic synapse” were the key signaling pathways in which the target genes of lncRNAs that changed between days 5 and 15 of gestation were expressed (Figure 3A). “Natural killer cell mediated cytotoxicity”, “Amphetamine addiction”, “Dopaminergic synapse”, “Necroptosis”, “Glutamatergic synapse”, and “Influenza A” were the primary complications between days 5 and 18 of pregnancy (Figure 3B). Target genes of differential lncRNAs were primarily involved in “Natural killer cell mediated cytotoxicity”, “Thyroid hormone signaling pathway”, “Amphetamine addiction”, and “Tryptophan metabolism” between 15 and 18 days of gestation (Figure 3C).

### 3.3. Validation of Deep Sequencing Results by RT-qPCR

**Figure 4:**
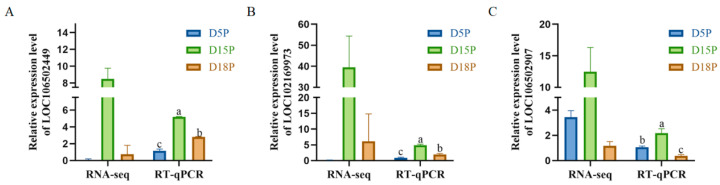
RT-qPCR validation of the differential expression of lncRNAs LOC106502449 (**A**), LOC102169973 (**B**), and LOC106502907 (**C**) in uterine cavity fluid exosomes. *GAPDH* was used as a reference gene for normalization. The data are expressed as the mean ± SEM of three replicates. Different letters indicate significant differences.

Three lncRNAs (LOC106502449, LOC102169973, LOC106502907) were randomly selected for RT-qPCR detection. The results were consistent with the sequencing data. Compared with D5P, the expression levels of LOC106502449 and LOC102169973 in exosomes of uterine flushing fluid were significantly up-regulated on D15P and D18P (Figure 4A,B, *p* < 0.05). Compared with the D5P group, the expression level of LOC106502907 was significantly increased in the D15P group but significantly decreased in the D18P group (Figure 4C, *p* < 0.05).

### 3.4. Construction of Co-Expression Network

**Figure 5:**
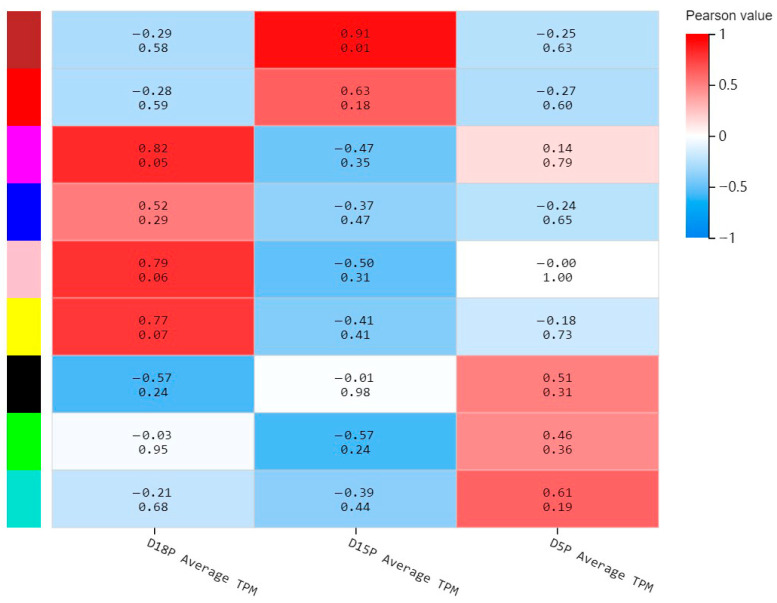
Weighted gene co-expression network analysis. The *x*-axis indicates the different groups, and the *y*-axis indicates the different modules. The number above each cell indicates the correlation between the module and the sample. Values closer to 1 signify a stronger positive correlation, while values closer to −1 signify a stronger negative correlation. The number below each cell represents the significance *p*-value, with smaller values indicating higher significance.

A total of 2974 differentially expressed mRNAs (DEMs) from the three gestational groups were extracted from our previous sequencing data and subjected to joint analysis with 100 differentially expressed lncRNAs (DELs) identified in this study. And nine co-expression modules were identified using WGCNA, which revealed the molecular gene regulatory networks based on pairwise correlations between the differentially expressed RNAs (Figure 5). The RNAs that might function together were considered as one module and assigned a single color. Notably, the blue-green module exhibited positive correlation with the D5P group but negative correlation with the D18P and D15P groups; the brown module showed positive correlation with the D15P group and negative correlation with the D18P and D5P groups; while the yellow module was positively associated with the D18P group and negatively correlated with the D15P and D5P groups (Figure 5). Key DELs/DEMs in these three modules were identified for further analysis.

### 3.5. Functional Enrichment Analysis

**Figure 6:**
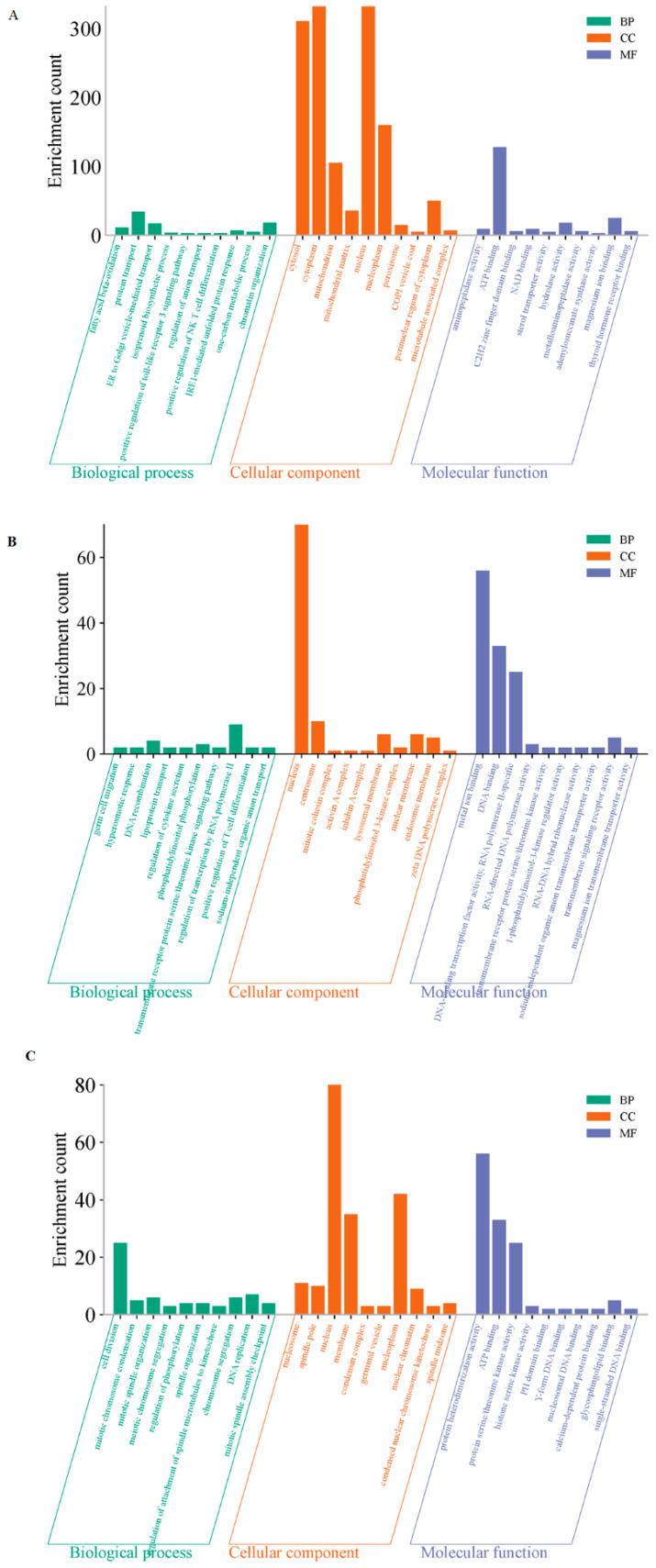
GO enrichment analyses of the three modules. (**A**) The blue-green module. (**B**) The brown module. (**C**) The yellow module. The *X*-axis represents enriched terms; the *Y*-axis represents the number of enriched genes. BP: biological process; CC: cellular component; MF: molecular function.

To explore the functional roles of these different modules, the GO and KEGG analyses were further performed. GO annotation revealed that the primary functions of these differentially expressed lnRNAs and mRNAs on D5P were “ATP binding” and “protein transport” (Figure 6A). The main functions of the RNA interacting on D15P were related to “DNA binding” and “metal ion binding” (Figure 6B). The enriched lnRNAs and mRNAs on D18P were mainly involved in “ATP binding”, “protein heterodimerization activity”, and “cell division” (Figure 6C).

**Figure 7:**
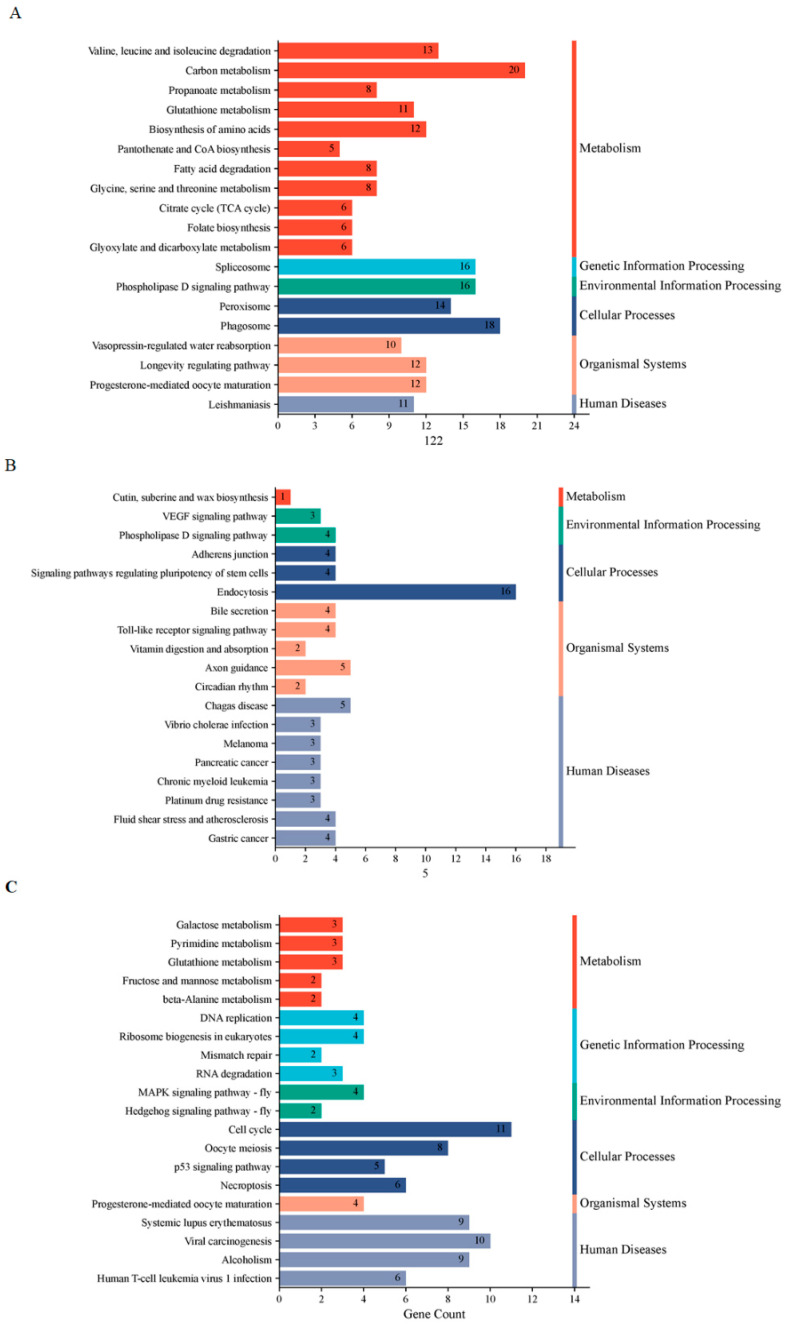
KEGG enrichment analyses of the three modules. (**A**) The blue-green module. (**B**) The brown module. (**C**) The yellow module.

For the KEGG pathway analysis, the most significantly enriched pathways at D5P included “carbon metabolism” and “phagosome” (Figure 7A). At D15P, key enriched pathways comprised “VEGF signaling pathway” and “endocytosis” among others (Figure 7B). By D18P, the enriched lncRNAs and mRNAs were mainly involved in “Cell cycle”, “Viral carcinogenesis” and “MAPK signaling pathway” (Figure 7C).

### 3.6. Construction of ceRNA Network

**Figure 8:**
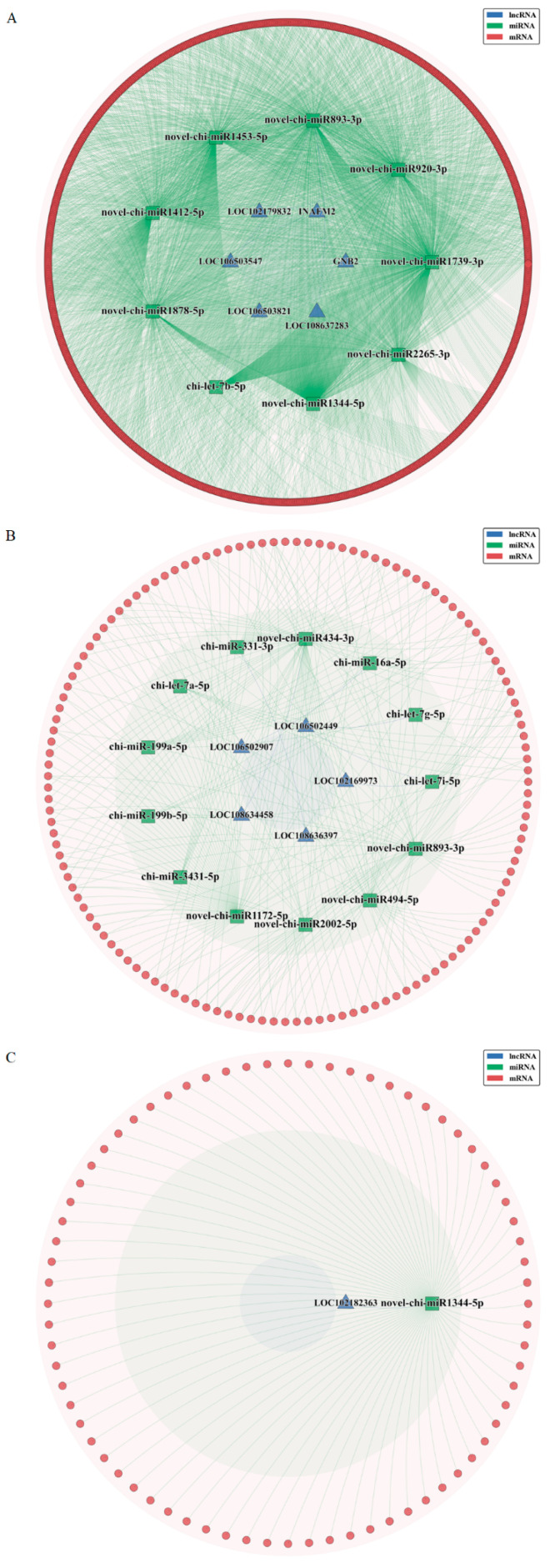
Construction of the competing endogenous RNA network. (**A**) The blue-green module. (**B**) The brown module. (**C**) The yellow module.

Based on the WGCNA co-expression modules, we further predicted interactions of lncRNAs/mRNAs with miRNAs and constructed a lncRNA-miRNA-mRNA ceRNA regulatory network (Figure 8). The predicted lncRNA-miRNA and mRNA-miRNA pairs for lncRNA-related ceRNA networks are detailed in Supplementary Tables S1–S3.

## 4. Discussion

The low reproductive rates in ruminants pose a significant challenge for the livestock industry. Enhancing the success rate of embryo implantation has emerged as a critical focus within the fields of reproductive and assisted reproductive technologies. Exosomes, which are membrane-bound vesicles secreted into extracellular fluids, contain a variety of biomolecules, including proteins, DNA, mRNAs, and lncRNAs [28,29,30]. Studies have shown that exosomes are present in almost all eukaryotic body fluids, including uterine fluid, urine, amniotic fluid, and breast milk [31]. They can participate in regulating various biological processes, such as immune modulation, cell proliferation, antigen presentation, and bioenergetic transformation [32]. Increasing evidence suggests that exosomes may be involved in regulating maternal responses, maintaining cellular metabolic homeostasis, promoting fetal angiogenesis and adapting maternal uterine vasculature during pregnancy [33,34,35]. Therefore, exosome-mediated crosstalk between the endometrium and the blastocyst during the pre-implantation stage and placental development is likely pivotal for pregnancy initiation. However, research on exosome lncRNAs during early pregnancy remains relatively scarce. In this study, we characterized the expression profiles of lncRNAs in exosomes isolated from goat uterine fluid on days 5, 15, and 18 of early pregnancy. Analyses revealed significant stage-specific differences in exosomal lncRNA profiles, suggesting their potential regulatory roles in embryo implantation.

LncRNA-mRNA regulatory system has been shown to play a crucial role in the successful implantation of embryos [17,36,37,38]. In this study, GO analysis revealed that the target genes of differentially expressed lncRNAs were primarily enriched in biological processes and pathways related to embryo implantation and endometrial development, such as signaling receptor activity, binding, and immune response. These processes are essential for the successful implantation of embryos [39,40,41]. A critical event required for embryonic development and pregnancy maintenance in mammals is maternal immune tolerance to the fetus. Studies have shown that exosomes derived from placental and uterine flushing fluids regulate the expression of immune-related genes and signaling pathways, thereby participating in maternal–fetal interface immune regulation [42,43]. Furthermore, KEGG analysis indicated that the target genes of the three groups of DELs were significantly enriched in the “Natural killer cell mediated cytotoxicity” signaling pathway, suggesting that exosomal lncRNAs play an important role in regulating pregnancy-related immunity. Additionally, the enrichment of pathways such as “Glutamatergic synapse” and “Dopaminergic synapse” further supports DELs’ involvement in intercellular signaling regulation, indicating their potential role in maternal–fetal communication.

Moreover, we employed WGCNA to construct nine functional modules and selected those significantly positively correlated with corresponding gestational days for in-deep analysis. Successful embryo implantation requires two essential conditions: a healthy embryo with implantation potential and uterus in a receptive state. Prior to implantation, nutrient delivery within the uterine cavity, coupled with sequential changes in endometrial morphology, tissue structure, and secreted proteins during the receptive phase, is crucial for successful implantation [44,45,46]. Our results indicate that differentially expressed lncRNAs and mRNAs on D5P are mainly involved in protein transport and metabolic processes; those at D15P are primarily associated with angiogenesis regulation, cell adhesion, signaling pathways, and immune-related pathways; while at D18P, differentially expressed lncRNAs and mRNAs are predominantly linked to biological events related to cell proliferation, apoptosis, and cellular metabolism. Collectively, embryo implantation is a spatiotemporally regulated biological process, and the dynamic changes in the molecular composition of exosomes from uterine washing fluid across stages corroborate this precise regulation.

LncRNAs can influence gene expression levels by acting as ceRNAs to bind miRNAs—which has been implicated in pregnancy progression and related pathologies. In this study, we identified and constructed ceRNA networks involving 12 lncRNAs across three groups: LOC102169973, LOC106502449, LOC106502907, LOC108634458, LOC108636397, LOC102182363, GNB2, INAFM2, LOC102179832, LOC106503547, LOC106503821, and LOC108637283. Although the functions of these lncRNAs have not been verified, GO and KEGG analyses suggest their potential involvement in the implantation process of goat embryos, which is worthy of further study.

## 5. Conclusions

In summary, through sequencing analysis of exosomes isolated from uterine flushing fluids of goats on days 5, 15, and 18 of gestation, we identified 831 differentially expressed lncRNAs. Functional enrichment analyses revealed that these lncRNAs are primarily involved in early-stage-pregnancy-associated biological processes, including cell communication and immune regulation. WGCNA further identified nine co-expression modules, and analyses of these modules demonstrated that lncRNAs and mRNAs within uterine flushing fluid exosomes exhibited dynamic temporal changes across distinct gestational stages. Additionally, we constructed ceRNA regulatory networks centered on key lncRNAs. These findings provide new insights into maternal–fetal communication during goat embryo implantation and establish a solid experimental foundation for screening and investigating uterine exosome-derived lncRNAs that modulate embryo implantation of goats.

## Figures and Tables

**Table 1:** Primer sequence for RT-qPCR.

Gene Name	Primer Sequences (5′–3′)
LOC106502449	F-TCTGATGACCTGCCCTCT R-CTCTGCCTCTTCTTCTGG
LOC102169973	F-ATAACTGACGGAAGACGA R-GTAGCGGACAAATAGGAG
LOC106502907	F-GGGCACCTTTTATTTCTA R-GCTAAGAGGCTTTTGGTC
*GAPDH*	F-TCTGCTGATGCCCCCATGTT R-TGACCTTGCCCACAGCCTTG

## Data Availability

The data presented in this study are openly available in the NCBI SRA under the BioProject accession number PRJNA1286071. [SRA] [https://www.ncbi.nlm.nih.gov/sra/PRJNA1286071] [PRJNA1286071].

## Supplementary Materials

The following supporting information can be downloaded at https://www.mdpi.com/article/10.3390/ani15172471/s1, Table S1: LncRNA-miRNA and mRNA-miRNA interaction pairs in lncRNA-associated ceRNA networks (blue-green module); Table S2: LncRNA-miRNA and mRNA-miRNA interaction pairs in lncRNA-associated ceRNA networks (brown module); Table S3: LncRNA-miRNA and mRNA-miRNA interaction pairs in lncRNA-associated ceRNA networks (yellow module).
